# Mitotic arrest-induced phosphorylation of Mcl-1 revisited using two-dimensional gel electrophoresis and phosphoproteomics: nine phosphorylation sites identified

**DOI:** 10.18632/oncotarget.12586

**Published:** 2016-10-12

**Authors:** Rong Chu, Sarah E. Alford, Katherine Hart, Anisha Kothari, Samuel G. Mackintosh, Matthew R. Kovak, Timothy C. Chambers

**Affiliations:** ^1^ Department of Biochemistry and Molecular Biology, University of Arkansas for Medical Sciences, Little Rock, AR 72205, USA

**Keywords:** Mcl-1, phosphorylation sites, mitotic arrest

## Abstract

Microtubule targeting agents (MTAs) characteristically promote phosphorylation and degradation of Mcl-1, and this represents a critical pro-apoptotic signal in mitotic death. While several phosphorylation sites and kinases have been implicated in mitotic arrest-induced Mcl-1 phosphorylation, a comprehensive biochemical analysis has been lacking. Contrary to previous reports suggesting that T92 phosphorylation by Cdk1 regulates Mcl-1 degradation, a T92A Mcl-1 mutant expressed in HeLa cells was phosphorylated and degraded with the same kinetics as wild-type Mcl-1 following vinblastine treatment. Similarly, when Mcl-1 with alanine replacements of all five putative Cdk sites (S64, T92, S121, S159, T163) was expressed, it was also phosphorylated and degraded in response to vinblastine. To analyze Mcl-1 phosphorylation in more detail, two-dimensional gel electrophoresis (2D-PAGE) was performed. While untreated cells expressed mainly unphosphorylated Mcl-1 with two minor phosphorylated species, Mcl-1 from vinblastine treated cells migrated during 2D-PAGE as a train of acidic spots representing nine or more phosphorylated species. Immunopurification and mass spectrometry of phosphorylated Mcl-1 derived from mitotically arrested HeLa cells revealed nine distinct sites, including several previously unreported. Mcl-1 bearing substitutions of all nine sites had a longer half-life than wild-type Mcl-1 under basal conditions, but still underwent phosphorylation and degradation in response to vinblastine treatment, and, like wild-type Mcl-1, was unable to protect cells from MTA treatment. These results reveal an unexpected complexity in Mcl-1 phosphorylation in response to MTAs and indicate that previous work has severely underestimated the number of sites, and thus encourage major revisions to the current model.

## INTRODUCTION

Mcl-1 is a pro-survival member of the Bcl-2 family of apoptotic regulatory proteins that plays a key role in the survival and development of diverse cell types [1]. Mcl-1 overexpression occurs in many tumor types and is a principal factor in chemoresistance, especially toward inhibitors of the other major pro-survival proteins, Bcl-2 and Bcl-xL [1–6]. Mcl-1 differs from Bcl-2 and Bcl-xL in that it lacks a BH4 domain and contains an extended N-terminal region [7]. This region includes several PEST sequences and sites for ubiquitination, phosphorylation, and caspase cleavage, all of which are important in regulating Mcl-1 stability [7]. In particular, site-specific phosphorylation has been shown to be a key mechanism controlling Mcl-1 expression level. For example, phosphorylation by ERK at T92 and T163 stabilizes Mcl-1 by prolonging its half-life [8], whereas phosphorylation by GSK3β at S159 acts as a signal for ubiquitination and subsequent proteosomal degradation [9]. Phosphorylation of other sites has also been reported and this alters the binding properties of Mcl-1 for its pro-apoptotic partners. For example, Mcl-1 is phosphorylated on S64 by CDK or JNK and this enhances its binding to Bak, Noxa, and Bim [10]. Data showing that stress-induced Mcl-1 degradation requires the activities of pro-apoptotic JNK and of GSK3, the latter inhibited by pro-survival AKT, emphasize the key role that Mcl-1 plays in coordinating death and survival signals [11].

We initially reported that microtubule targeting agents^3^ (MTAs), including vinblastine, paclitaxel, and vincristine, induced Mcl-1 phosphorylation and degradation in KB3 cells [12, 13]. Additional work from our laboratory [13–16] and independent studies from several other laboratories [17–19] have shown that this is a general response to mitotic arrest in a wide variety of cell lines. In KB3 cells, a key role for Mcl-1 is to sequester Bak, and MTA-induced phosphorylation and degradation results in release and activation of Bak [13]. The relevant sites of phosphorylation and corresponding protein kinases responsible have been the subject of several studies. It was reported that the major sites of phosphorylation in nocodazole-treated HeLa cells were T92 and S64, and that Mcl-1 destruction during mitotic arrest was initiated by phosphorylation of T92 catalyzed by Cdk1/cyclin B1 and subsequent ubiquitination by APC/C^Cdc20^ [17]. In mitotically arrested HCT116 cells expressing FLAG-Mcl-1, phosphorylation sites were identified as T92, S121, S159 and T163, with Cdk1, p38, CKII and JNK implicated as key protein kinases [19]. Based on these results, a model was proposed whereby Cdk1 phosphorylates T92, displacing bound protein phosphatase PP2A, allowing net phosphorylation at S121, S159, and T163, mediated by p38, CKII, and JNK, respectively, which in turn allowed recruitment of SCF^FBW7^, resulting in Mcl-1 degradation [19]. Thus, while Cdk1 is hypothesized to play a key priming role to initiate Mcl-1 phosphorylation during mitotic arrest, the nature of the ubiquitin ligase system responsible for subsequent degradation is uncertain.

In this study, two-dimensional gel electrophoresis (2D-PAGE) and phosphoproteomics were employed to analyze phosphorylation of wild-type and phospho-mutant forms of Mcl-1 expressed in HeLa cells. Our results argue against a priming role for Cdk1, show that mitotic arrest-induced Mcl-1 phosphorylation is far more complex than previously suspected, and encourage revisions to current models.

## RESULTS

### Mcl-1 is highly phosphorylated during mitotic arrest

**Figure 1:**
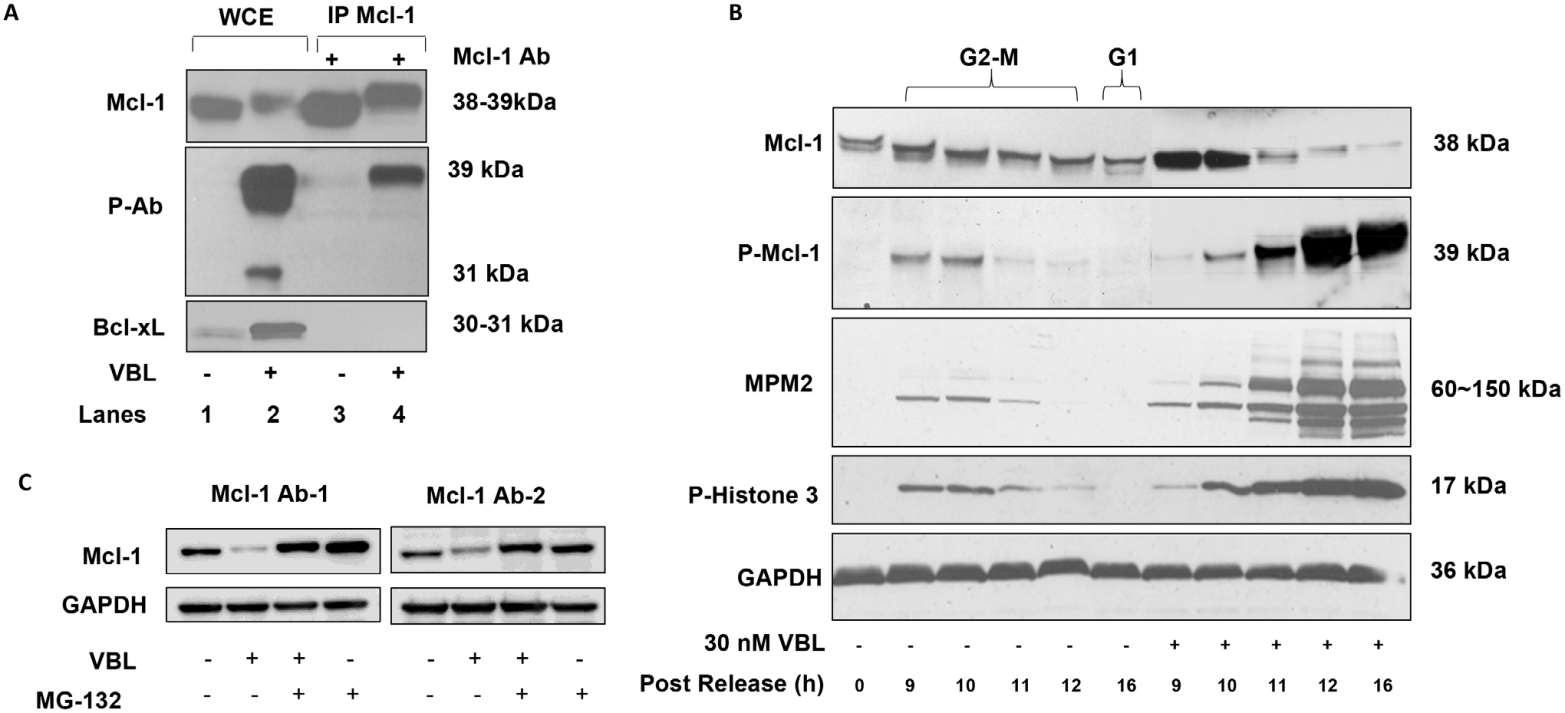
Validation of phospho-specific Mcl-1 antibody, kinetics of Mcl-1 phosphorylation in response to vinblastine, and effect of MG132 **A.** HeLa cells were untreated or treated with vinblastine (VBL) as indicated and whole cell extracts (WCE, lanes 1 and 2) or immunoprecipitated (IP) Mcl-1 (lanes 3 and 4) subjected to immunoblotting for Mcl-1 (upper panel) or with a phospho-specific antibody (p-Ab) originally developed to detect phospho-Bcl-xL (middle panel) or with a phosphorylation-independent Bcl-xL antibody (lower panel). **B.** HeLa cells were synchronized at the G1/S boundary by double thymidine block and were either untreated or treated with 30 nM vinblastine (VBL) 1 h after release, then harvested at the indicated times. In the untreated group, time points corresponding to late G2-M and G1 phases are indicated. Extracts were subjected to immunoblotting for the proteins indicated, with GAPDH used as a loading control. Phosphorylated Mcl-1 (P-Mcl-1) was detected with an antibody developed to detect phosphorylated Bcl-xL as in Figure 1A. **C.** HeLa cells were treated with 30 nM vinblastine (VBL) for 24 h, in the presence or absence of 20 μM MG132 added 4 h prior to harvest, and extracts prepared and subjected to immunoblotting for Mcl-1. In the left panel (Mcl-1 Ab-1), Mcl-1 antibody sc-12756 was used, and in the right panel (Mcl-1 Ab-2), Mcl-1 antibody sc-20679 was used.

As we reported previously [13], Mcl-1 undergoes a phosphorylation-induced mobility shift and degradation after vinblastine treatment of HeLa cells (Figure 1A, upper panel, lanes 1 and 2). In order to facilitate analysis of Mcl-1 phosphorylation we sought to identify an antibody which recognized phosphorylated Mcl-1 from mitotically arrested cells. Previously we had utilized a commercially available antibody against S62-phosphorylated Bcl-xL, which recognized phosphorylated Bcl-xL (31 kDa) in extracts from vinblastine–treated HeLa cells [15]. We discovered that this antibody, in addition to recognizing phosphorylated Bcl-xL at 31 kDa, also strongly reacted with a protein of 39 kDa in extracts from vinblastine-treated HeLa cells but not in control extracts (Figure 1A, middle panel, lanes 1 and 2). The 39 kDa protein migrated at a similar position to phosphorylated Mcl-1 observed with the phosphorylation-independent antibody (Figure 1A, upper panel, lane 2). Immunoprecipitation of Mcl-1 from the same samples was performed and Mcl-1 was readily precipitated from both the control and vinblastine-treated cell extracts (Figure 1A, upper panel, lanes 3 and 4). Phosphorylated Mcl-1 immunoprecipitated from vinblastine-treated cells was recognized by the phospho-specific antibody (Figure 1A, middle panel, lane 4), confirming its identity as Mcl-1, whereas phosphorylated Bcl-xL was not detected in the Mcl-1 immunoprecipitate, as expected. When the samples were probed for Bcl-xL (Figure 1A, lower panel), Bcl-xL was observed in the whole cell extracts as expected but not in the Mcl-1 immunoprecipitates, further demonstrating Mcl-1 antibody specificity. Note that with the phosphorylation-independent antibody, phosphorylated (shifted) Bcl-xL reacts more strongly than unphosphorylated Bcl-xL, as we have observed previously [20]. When mock immunoprecipitations were conducted in the absence of antibody, no immunoreactive bands were observed (data not shown). The phospho-specific antibody was next used to assess the status of Mcl-1 phosphorylation in synchronized HeLa cells traversing mitosis without or with vinblastine treatment. As shown in Figure 1B, a band of 39 kDa reactive with the phospho-specific antibody was observed at 9-10 h after release in the absence of vinblastine treatment. Bands corresponding to phospho-H3 histone and reactive with MPM2 antibody, which recognizes mitotic phosphoproteins, were observed with a similar time-course. These results indicate that Mcl-1 is transiently phosphorylated during mitosis, and are consistent with previous results that HeLa cells enter and traverse M phase approximately 9-10 h after release from double thymidine block [21]. Cells were then released in the presence of vinblastine to induce mitotic arrest, confirmed by strong and persistent phospho-H3 and MPM2 staining (Figure 1B). Under these conditions, Mcl-1 underwent a mobility shift and degradation, and corresponding bands reactive with the phospho-specific antibody were observed. The fact that strong immunoreactivity with the phospho-specific antibody was observed concomitant with relatively low levels of total Mcl-1 suggests a very high level of phosphorylation occurs under these conditions, a conclusion supported by subsequent experiments described below. To exclude the possibility that loss of total Mcl-1 expression was an artifact due to reduced affinity of the antibody for highly phosphorylated Mcl-1, we treated cells with vinblastine in the absence or presence of the proteosome inhibitor, MG132. As shown in Figure 1C, MG132 blocked vinblastine-induced loss of Mcl-1 expression, and this was observed with two independent antibodies recognizing different Mcl-1 motifs. This confirms that the loss of Mcl-1 expression was due to protein degradation and was not an artifact resulting from reduced antibody affinity.

### Phosphorylation of Mcl-1 by Cdk1 is dispensable for vinblastine-induced degradation

**Figure 2:**
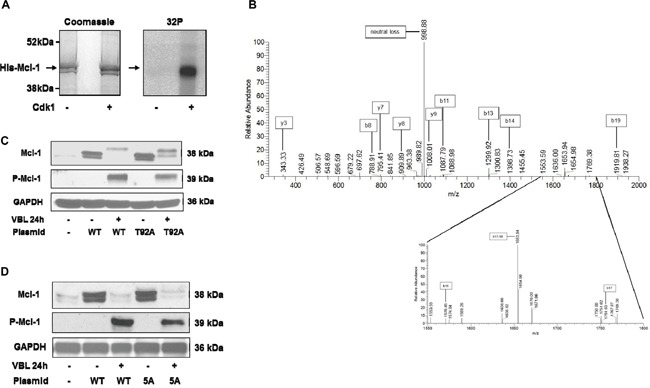
Phosphorylation of Mcl-1 on T92 by Cdk1 and analysis of Mcl-1 harboring mutations of Cdk consensus sites **A.** His-tagged recombinant Mcl-1 was incubated under phosphorylation conditions with [γ-^32^P]ATP either in the absence or presence of purified active Cdk1/cyclin A2, as indicated, as described in Materials and Methods. Samples were resolved by SDS-PAGE and stained (Coomassie) and subjected to phosphor-image analysis (^32^P), as indicated. **B.** Identification of major phosphorylation site as T92. MS/MS spectrum of phosphorylated Mcl-1 peptide VARPPPIGAEVPDVTApTPAR (2093.07 Da monoisotopic molecular weight) with prominent b and y ions and neutral loss product indicated. The peak at 998.88 m/z is consistent with neutral loss of 79.97 Da characteristic of phosphorylation. The b16, b17-98, and b17 ions shown in the detail from 1550-1800 m/z indicate phosphorylation of Thr92. **C, D.** HeLa cells stably overexpressing wild-type (WT) or T92A (C) or 5A (D) Mcl-1 were untreated or treated with 30 nM vinblastine (VBL) for 24 h, and extracts immunoblotted for the proteins indicated. Parental HeLa cells are shown in the left lanes of each panel; endogenous Mcl-1 is only weakly detected under these conditions. GAPDH was used as a loading control.

Several reports have indicated that Cdk1/cyclin B1 phosphorylates Mcl-1 at T92 during mitotic arrest thus priming it for additional phosphorylation events [17, 19]. Incubation of recombinant Mcl-1 with purified active Cdk1 under phosphorylation conditions confirmed Mcl-1 as an *in vitro* substrate (Figure 2A). To identify site(s) of Cdk1 phosphorylation, excised gel bands were processed and subject to mass spectrometric analysis, as described in Materials and Methods. As shown in Figure 2B, the MS/MS spectrum of phosphorylated Mcl-1 peptide (VARPPPIGAEVPDVTApTPAR) unambiguously indicate phosphorylation of T92. The peak at 998.88 m/z is consistent with neutral loss of 79.97 Da characteristic of phosphorylation. The b16, b17-98, and b17 ions shown in the detail from 1550-1800 m/z localize phosphorylation of the peptide to T92. Supplementary Figure S1 shows the MS/MS spectrum of the unphosphorylated peptide (VARPPPIGAEVPDVTATPAR), derived from the reaction conducted in the absence of Cdk1 (Figure 2A), for comparison. The b and y ion series are indicative of the same peptide sequence without the mass shift and neutral loss signatures characteristic of phosphorylation.

HeLa cells overexpressing wild-type and T92A mutant Mcl-1 were generated and treated with vinblastine. As show in Figure 2C, T92A mutant Mcl-1 underwent phosphorylation and degradation similar to wild-type. Furthermore, when Mcl-1 with alanine mutations of all five putative Cdk1 (proline-directed) phosphorylation sites (S64, T70, T92, S121, T163) (5A) was expressed, it too was phosphorylated and degraded (Figure 2D). These results suggest that Cdk1-mediated phosphorylation is dispensable for mitotic arrest-induced Mcl-1 phosphorylation/degradation, and prompted more detailed biochemical analysis.

### Analysis by two-dimensional gel electrophoresis

**Figure 3:**
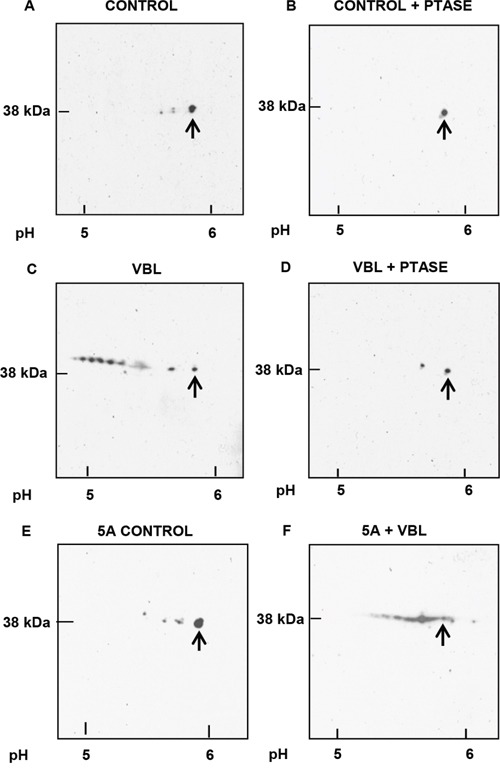
Analysis of Mcl-1 phosphorylation by 2D-PAGE Samples were prepared for analysis as described in Materials and Methods with isoelectric focusing from right to left and SDS-PAGE from top to bottom. Approximate pH values are shown at the bottom, and arrows indicate unphosphorylated Mcl-1. **A.** Asynchronous (control) HeLa cells. **B.** As in A, except that extract was treated with lambda phosphatase (PTASE) before analysis. **C.** Vinblastine (VBL) treated: HeLa cells were synchronized at the G1/S boundary by double thymidine block, treated with 30 nM VBL 1 h after release, and harvested during mitotic arrest 12 h post-release, with the final 2 h in the presence of 20 μM MG132. **D.** As in C, except that the extract was treated with PTASE. **E.** Asynchronous HeLa cells expressing the 5A Mcl-1 mutant. **F.** HeLa cells expressing the 5A mutant and synchronized and treated with VBL, as in C.

To examine mitotic arrest-induced Mcl-1 phosphorylation in more detail, two-dimensional gel electrophoresis (2D-PAGE)/immunoblotting was employed. In preliminary experiments using standard conditions of sample preparation where proteins such as Bcl-2 or Bcl-xL were readily detected [22], we failed to detect Mcl-1 on the gels after immunoblotting (data not shown). Several different procedures were tested and it was found that the technique of Herbert et al. [23], which improves protein solubility though the use of tributyl phosphine, provided conditions for the detection of Mcl-1. Thus, asynchronous control cells showed one major and two minor, more acidic, spots, the latter sensitive to acid phosphatase treatment (Figure 3A, 3B). Extracts from mitotically arrested cells showed a train of acidic spots that varied in independent experiments from eight to nine distinct species, all but one of which was eliminated by acid phosphatase (Figure 3C, 3D). Whether the non-eliminated spot represents an acidic modification distinct from phosphorylation, or a phospho-species resistant to dephosphorylation, is unclear. The pI of the most acidic form of Mcl-1 was 4.9 (Figure 3C), corresponding closely to the calculated pI of 4.84 for Mcl-1 containing nine phosphates (www.phosphosite.org). Importantly, mitotic cells showed only three phosphorylated Mcl-1 species (data not shown), indicating that mitotic arrest promotes the phosphorylation of many additional sites. After vinblastine treatment of cells expressing the 5A mutant, several phosphorylated species were observed (Figure 3E), but the pattern was much simpler than that of the wild-type protein, consistent with the absence of five sites present in the wild-type, and consistent too with the presence of several non-proline directed sites. These results indicate that Mcl-1 undergoes phosphorylation on a large number of sites during mitotic arrest, including many that are not normally phosphorylated during mitosis and including several that are not proline-directed.

### Identification of mitotic arrest-associated Mcl-1 phosphorylation sites

**Figure 4:**
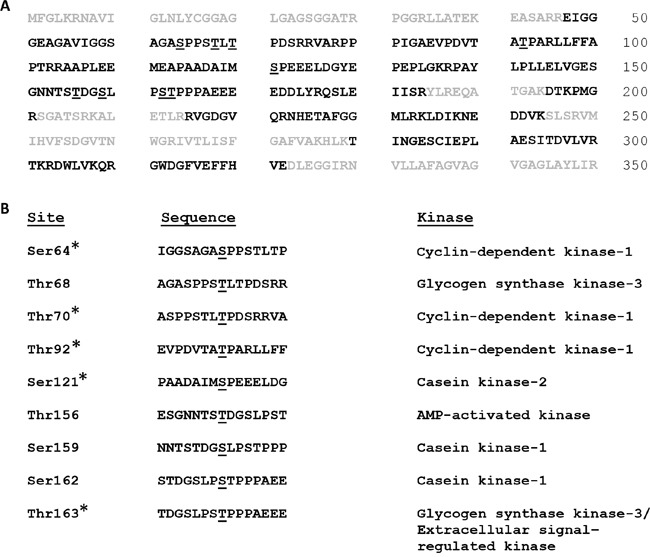
Mcl-1 phosphorylation sites associated with mitotic arrest **A.** Immuno-purified Mcl-1 was digested with trypsin and peptides were analyzed by MS/MS, as described in Methods and Materials and Supplementary Figure S2, which identified the peptide sequences shown in bold representing 63% coverage. Sites of phosphorylation are underlined. **B.** Sites of phosphorylation, sequence context, and predicted kinases based on matches to established recognition motifs. Sites marked with an asterisk were manually validated. See text for details.

In order to identify the sites of Mcl-1 phosphorylation associated with mitotic arrest, Mcl-1 was purified by preparative immunoprecipitation from synchronized, vinblastine treated HeLa cells, as described in Materials and Methods. Because this type of phosphorylation leads to ubiquitination and proteosome-mediated degradation [17, 19] (Figure 1C), the proteosome inhibitor MG132 was added 2 h before harvest to protect Mcl-1. Analysis of the preparative Mcl-1 immunoprecipitate by SDS-PAGE and Coomassie Blue staining is shown in Supplementary Figure S2A. Several prominently staining bands were evident which were presumed to be protein A/G and light and heavy chains from IgG. A stained band of the expected molecular mass of ∼40 kDa was observed (marked by an asterisk in Supplementary Figure S2A), excised, and subjected to tandem MS (MS/MS) using an LTQ Orbitrap Velos mass spectrometer, as described in Materials and Methods. To identify peptides, a UniProt database search using Mascot was run, and Scaffold was then used to validate MS/MS-based identifications. Peptide identifications were accepted if they were scored at ≥ 95% probability by the Scaffold local false-discovery rate (FDR) algorithm. Peptides corresponding to Mcl-1 were observed, with overall sequence coverage of 63% (Figure 4A). MS/MS spectra corresponding to phosphopeptides were also manually validated for unambiguous site localization. Nine phosphorylation sites meeting the FDR algorithm threshold were identified; these were S64, T68, T70, T92, S121, T156, S159, S162, T163 (Figure 4A, 4B). Five of these sites were manually validated (Figure 4B); the corresponding MS/MS spectra are shown in Supplementary Figure S2, panels B-H. Importantly, the total number of sites identified through MS analysis was consistent with data derived from 2D-PAGE analysis. Furthermore, the same nine sites were predicted when the human Mcl-1 sequence was scanned for phospho-motifs (http://scansite3.mit.edu). The kinase(s) whose recognition motifs best match each site is shown in Figure 4B, and similar information was obtained by searching other kinase/phospho-motif databases. Three of the sites are novel and, to our knowledge, have not been reported previously (T68, T70, T156), and the others are established with various kinases ascribed to them [7], with the exception of S162 which was more recently reported [24].

### Characterization of 9A phospho-mutant Mcl-1

**Figure 5:**
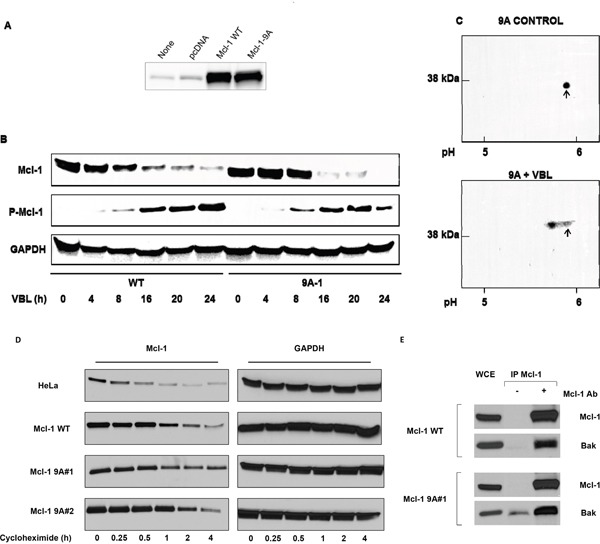
Vinblastine-induced phosphorylation and degradation, protein stability, and Bak binding properties, of Mcl-1 9A mutants **A.** HeLa cells were untransfected, or transfected with vector or with plasmids encoding wild-type (WT) or Mcl-1-9A (with replacements of all nine phosphorylation sites, see Figure 4), and extracts subjected to immunoblotting for Mcl-1. **B.** Time-course of VBL treatment in HeLa cells expressing WT or Mcl-1-9A. Cells were treated with 30 nM VBL for the times indicated and extracts subjected to immunoblotting for the indicated proteins. **C.** HeLa cells expressing Mcl-1-9A were untreated (control) or treated with 30 nM VBL for 16 h. Samples were prepared for 2D-PAGE analysis as described in Materials and Methods with isoelectric focusing from right to left and SDS-PAGE from top to bottom. Approximate pH values are shown at the bottom, and arrows indicate unphosphorylated Mcl-1. **D.** Parental HeLa cells, or cells overexpressing wild-type (WT) or 9A mutant Mcl-1 (two independent clones) were treated with 1 μg/mL cycloheximide for the times indicated, and extracts subjected to immunoblotting for Mcl-1 or GAPDH as indicated. **E.** Both wild-type and 9A mutant Mcl-1 interact with Bak. Whole cell extracts (WCE) from HeLa cells overexpressing wild-type (WT) or 9A mutant Mcl-1 were immunoprecipitated with Mcl-1 antibody, and subjected to immunoblotting for either Mcl-1 or Bak, as indicated. Mock immunoprecipitations conducted in the absence of Mcl-1 antibody are also shown.

A plasmid encoding Mcl-1 harboring alanine mutations of the nine sites shown in Figure 4 was created, and mutations were verified by sequence analysis. Several clones of HeLa cells stably overexpressing 9A mutant Mcl-1 (Mcl-1-9A) were prepared, and chosen for further study based on exhibiting comparable levels of overexpression versus wild-type Mcl-1. Results using a representative clone are presented in Figure 5A–5C; similar results were obtained with two other clones. Mcl-1 expression in cells stably overexpressing wild-type or Mcl-1-9A is shown in Figure 5A. A time-course of vinblastine treatment confirmed loss of Mcl-1 expression with a concomitant increase in phospho-Mcl-1 level in cells overexpressing wild-type Mcl-1 (Figure 5B), similar to findings with endogenous Mcl-1 (Figure 1). Essentially the same results were found for cells overexpressing Mcl-1-9A; the overall kinetics, and degree of reduction in expression of Mcl-1 and increase in phospho-Mcl-1 levels, were highly similar (Figure 5B). Collectively, these results strongly suggested that Mcl-1-9A, despite mutation of multiple identified phosphorylation sites, still undergoes vinblastine-induced phosphorylation and degradation. To further test this possibility, cells expressing Mcl-1-9A were untreated or treated with vinblastine, and extracts subjected to 2D-PAGE. As shown in Figure 5C, Mcl-1-9A from control cells resolved as a single spot which underwent a discrete shift to a more acidic form after vinblastine treatment, consistent with phosphorylation of the mutated protein possibly at a single additional site.

Other studies have indicated that certain phosphorylation sites, including T92 and T163 which are among the sites mutated in Mcl-1-9A, regulate Mcl-1 stability and prolong its half-life [7, 8]. To determine whether mutation of the identified phosphorylation sites altered Mcl-1 stability, parental HeLa cells, cells overexpressing wild-type Mcl-1, or two independent clones overexpressing Mcl-1-9A, were treated with cycloheximide to block protein synthesis, and at intervals up to 4 h, extracts were prepared for the analysis of Mcl-1 by immunoblotting. The results from a representative experiment are shown in Figure 5D. It is evident that the 9A mutants exhibit more persistent expression over this time-course. In order to quantitate the results, the experiment was repeated twice, and band intensity determined, as described in Methods and Materials, which was plotted over time to determine the half-life of Mcl-1 in each case. The following values, given as mean ± S.D., were obtained: endogenous Mcl-1, 26 ± 4 min; wild-type Mcl-1, 50 ± 10 min; 9A-1, 113 ± 9 min; and 9A-2, 118 ± 4 min. Thus mutation of the major phosphorylation sites significantly increased Mcl-1 half-life (p ≤ 0.002, WT vs. 9A-1 or 9A-2).

We have previously shown that an important role for Mcl-1 in HeLa cells is to bind and sequester the pro-apoptotic effector protein Bak [13]. To determine whether mutation of the nine phosphorylation sites altered this property, Mcl-1 was immunoprecipitated from cells overexpressing wild-type or 9A mutant Mcl-1, and immunoblotting performed for Mcl-1 and Bak. As shown in Figure 5E, Bak co-immunoprecipitated with both wild-type and 9A mutant Mcl-1. Quantitation of the results indicated that the ratio of Mcl-1 to Bak was essentially identical in each case (data not shown). Because vinblastine treatment causes Mcl-1 degradation, quantitative assessment of the presence of Bak in Mcl-1 immunoprecipitates from vinblastine treated cells was not possible.

**Figure 6:**
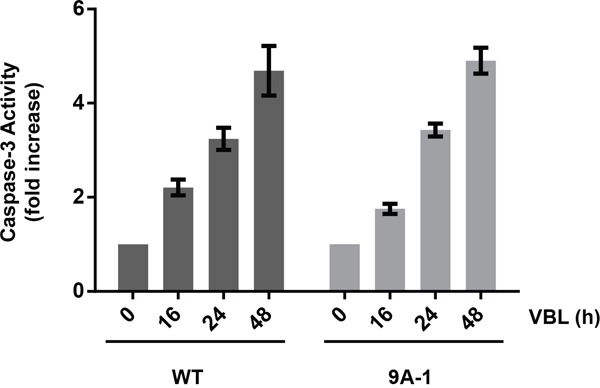
Caspase-3 activation in HeLa cells expressing Mcl-1-9A HeLa cells expressing wild-type (WT) or Mcl-1-9A were treated with 30 nM vinblastine (VBL) for the indicated times, and extracts subjected to caspase-3 assay, as described in Materials and Methods. Results are shown as fold-activation relative to untreated cells, and represent mean ± S.D. (n = 6).

To determine whether expression of Mcl-1-9A affected vinblastine sensitivity, several clones of HeLa cells stably overexpressing the protein (Supplementary Figure S3), were subjected to cell viability assays after vinblastine treatment, as described in Materials and Methods. While there was some variation in sensitivity, the IC_50_ values obtained were similar in general to that of cells expressing wild-type Mcl-1 and similar to parental HeLa cells (Table 1). Importantly, we have previously shown that wild-type Mcl-1 when transduced into leukemia cells confers resistance to the BH3 mimetics ABT-263 and ABT-199 [25]. Thus in the appropriate context, when the protein remains stable after drug challenge, it confers resistance as expected, and the lack of effect in cells treated with vinblastine can be attributed to the accompanying loss of expression. Furthermore, caspase-3 assays were conducted, and the kinetics and degree of activation of caspase-3 were similar for cells expressing either wild-type or Mcl-1-9A (Figure 6). These results indicate that expression of 9A Mcl-1 does not alter vinblastine sensitivity of HeLa cells.

**Table 1:** Vinblastine sensitivity of parental HeLa cells and HeLa cells expressing wild-type or 9A mutant Mcl-1

Cell Line	IC_50_(nM)
HeLa	0.40 ± 0.05
Mcl-1 WT	0.31 ± 0.02
Mcl-1 9A #1	0.32 ± 0.04
Mcl-1 9A #2	0.67 ± 0.21
Mcl-1 9A #3	0.37 ± 0.04
Mcl-1 9A #4	0.60 ± 0.09

IC_50_ values were determined as described in Materials and Methods. Data shown are mean ± S.D. (n = 6).

## DISCUSSION

Mcl-1 is subject to several different types of phosphorylation during the cell cycle and in response to various cellular stimuli which alter the stability and properties of this key pro-survival Bcl-2 family member. During normal mitosis two Cdk1-regulated sites have been reported; S64 [10, 17], the phosphorylation of which appears to enhance Mcl-1 pro-survival function [10], and T92, the phosphorylation of which may trigger Mcl-1 degradation [17]. Because mitotic T92 phosphorylation is transient and incomplete, cells can enter and exit mitosis keeping Mcl-1 largely intact and can thus divide successfully without approaching a death threshold [26]. The most dramatic changes in Mcl-1 phosphorylation occur during mitotic arrest, the consequence of which is widespread Mcl-1 degradation [27]. Indeed, Mcl-1 phosphorylation/degradation has been observed in a many cell lines during prolonged mitotic arrest and thus appears to be a universal and conserved response [16]. Existing models of mitotic arrest-induced Mcl-1 phosphorylation [27] suggest that T92 phosphorylation is required, either alone or in concert with phosphorylation of S121, S159, and T163, to trigger Mcl-1 degradation.

The results presented here indicate that current models are incomplete and underestimate the number of sites and in turn the complexity of mitotic arrest-induced Mcl-1 phosphorylation. In addition, contrary to the current paradigm, our results indicate that T92 phosphorylation is not required during mitotic arrest to trigger Mcl-1 phosphorylation and degradation. We identified nine sites of phosphorylation in this context, derived from several independent lines of investigation, including 2D-PAGE, tandem MS/MS, and a database search for phospho-motifs. Each of these approaches gave consistent results with respect to the high number and identity of the phosphorylation sites. Analysis by 2D-PAGE of wild-type Mcl-1 from vinblastine treated cells revealed a complex pattern of phospho-species, the most acidic of which exhibited a pI corresponding to Mcl-1 with nine phosphates (Figure 3C), further reinforcing this conclusion. Analysis of a 5A mutant (Mcl-1 lacking all proline-directed S/T sites), revealed a much simpler but still fairly complex pattern after vinblastine treatment (Figure 3F), confirming the presence of additional, non-proline directed sites. The 9A mutant, lacking all identified phospho-acceptor sites, underwent a discrete incremental acidic shift on 2D-PAGE after vinblastine treatment (Figure 5D) This confirmed that the mutated sites were indeed those phosphorylated endogenously to give rise to the complex train of acidic spots observed with wild-type Mcl-1. That the 9A mutant underwent vinblastine-induced phosphorylation suggests the existence of at least one site in addition to those identified by MS/MS. Several serine and threonine residues are present in the portion of sequence not covered by MS/MS analysis (Figure 4), and thus an additional phosphorylation site may have remained undetected. Alternately, there may be a certain degree of redundancy among the sites of phosphorylation, such that when preferred sites are absent, secondary sites become available for phosphorylation. In the latter case, secondary sites would escape detection upon analysis of wild-type Mcl-1.

Phosphorylation has been widely regarded as a key mechanism triggering Mcl-1 ubiquitination and subsequent degradation during mitotic arrest [26, 27]. In order to rigorously test this hypothesis, a phosphorylation-defective Mcl-1 mutant is needed. The 9A mutant described here is severely under-phosphorylated versus wild-type Mcl-1, but still retains the ability to become phosphorylated and degraded during mitotic arrest. If phosphorylation of the 9A mutant is indeed the trigger for its degradation, this again implies redundancy among the sites. Alternately, phosphorylation may be one pathway leading to Mcl-1 destruction during mitotic arrest, but there may be other pathways. In this regard, the role of Mcl-1 ubiquitination in the regulation of Mcl-1 expression and degradation is instructive. Using a mutant lacking the lysine residues required for ubiquitination, it was observed that murine Mcl-1 degradation by the proteasome can occur in the absence of ubiquitination [28]. Assuming the same holds true for the human homolog, and since the major role of phosphorylation is to promote ubiquitination, it follows that phosphorylation of Mcl-1 may also be dispensable for its degradation during mitotic arrest. Experiments with a completely phospho-defective Mcl-1, which has yet to be described, will be needed to answer this question. Although the lack of many major phosphorylation sites did not alter the ability of Mcl-1 to undergo vinblastine-induced degradation, the absence of these sites did significantly prolong Mcl-1 half-life under basal conditions (Figure 5D). The value of 24 min we obtained for the half-life of endogenous Mcl-1 is similar to a previous study, which reported a value of 40 min [29], emphasizing that Mcl-1 is a short-lived protein.

Many different protein kinases have been reported to phosphorylate Mcl-1; these include GSK3, JNK, ERK, p38, Cdk1/cyclin B1, and CK2 [7]. Recently, evidence was presented that Cdk2/cyclin E also phosphorylates and stabilizes Mcl-1 [30]. In this study, several different protein kinases were predicted to be responsible for the phosphorylation of the sites in Mcl-1 we identified (Figure 4B). For the sites already described in the literature, the identifications in general gave a corresponding match. For example, S64 and T92 were predicted in this study to be phosphorylated by Cdk1, and T163 by GSK3 or ERK, consistent with earlier reports [7]. We identified several sites which had not been previously reported; these included T68, T70, and T156. Interestingly, T156 was predicted to be phosphorylated by AMPK, which is the first preliminary indication that this kinase may play a role in Mcl-1 phosphorylation. These results broaden the number of kinases potentially involved in Mcl-1 phosphorylation.

In previous studies we have found that HeLa cells are critically dependent on Mcl-1 for survival, and if Mcl-1 levels are depleted using siRNA knockdown, the cells die via apoptosis [31]. However, overexpression of wild-type Mcl-1 in HeLa cells does not increase resistance to vinblastine and other inducers of mitotic arrest, whereas overexpression of Bcl-2 or Bcl-xL does impart resistance [15]. The basis for this observation appears to be that vinblastine causes loss of Mcl-1 expression via degradation, whereas Bcl-2 and Bcl-xL remain stably overexpressed after treatment. In this report we have confirmed this result, showing that parental HeLa cells and cells overexpressing Mcl-1 have similar degrees of sensitivity to vinblastine in cell viability assays (Table 1). Similarly, cells overexpressing Mcl-1-9A exhibit sensitivity to vinblastine in the same concentration range (Table 1). That Mcl-1-9A is subject to degradation like wild-type Mcl-1 is likely the reason that cells overexpressing this mutant are not more resistant to vinblastine. The generation and expression of a degradation resistant Mcl-1 will be needed to test the effect of Mcl-1 overexpression on cellular sensitivity to inducers of mitotic arrest. Given that redundant pathways likely exist for Mcl-1 degradation during mitotic arrest, this may be challenging to accomplish.

## MATERIALS AND METHODS

### Materials

Antibodies to Mcl-1 (sc-12756 and sc-20679) and Protein A/G PLUS-Agarose Beads (sc-2003) were from Santa Cruz Biotechnology (Dallas, TX); antibody to phospho-Bcl-xL (ab30655) and full length His-tagged human Mcl-1 protein (ab131682) were from Abcam (Cambridge, MA); antibodies to Bcl-xL (2762S), (phospho-histone H3 (9701S) and GAPDH (2118S) were from Cell Signaling Technology (Danvers, MA); purified active Cdk1/cyclin A2 was obtained from SignalChem (Richmond, BC, Canada); [γ-^32^P]ATP (BLU002A250UC) was from PerkinElmer (Waltham, MA); lambda protein phosphatase (P0753S) was from New England BioLabs (Ipswich, MA); OmniCleave^™^ endonuclease was from Epicentre (Madison, WI); tributylphosphine (T7567-10VL) and cycloheximide (C104450) were from Sigma-Aldrich (St. Louis, MO); Bio-Lyte 5/7 Ampholytes (163-1112) were from Bio-Rad (Hercules, CA); and porcine sequencing grade modified trypsin was from Promega (Madison, WI).

### Cell culture and synchronization

The HeLa human carcinoma cell line was maintained in monolayer culture at 37°C in 5% CO_2_ in Dulbecco's modified Eagle's medium supplemented with 10% fetal bovine serum, 2 mM L-glutamine, 50 U/mL penicillin, and 50 g/mL streptomycin. Cell line clones overexpressing Mcl-1 or phospho-mutants were prepared by transfecting HeLa cells with untagged human Mcl-1 in pcDNA3.1 vector followed by selection and maintenance in media containing 0.5 mg/mL G418, as described [22]. Cells were synchronized by double thymidine block method as described previously [32]. Briefly, 10^6^ cells in a 100-mm dish were incubated in medium containing 2 mM thymidine for 16 h, released into normal medium for 9 h, and then incubated for 16 h in medium containing 2 mM thymidine. To assess Mcl-1 protein stability, cells were treated with 1 μg/mL cycloheximide for intervals up to 4 h, and extracts prepared for immunoblotting.

### Preparation of cell extracts and immunoblotting

Whole cell extracts were prepared by suspending HeLa cells in 0.25 mL of lysis buffer (40 mM HEPES [pH 7.4], 120 mM NaCl, 1% Triton X-100, 50 mM NaF, 1 mM EDTA, EDTA-free complete protease inhibitor tablets, 1% aprotinin, 50 μg/ml leupeptin, 10 μM pepstatin, 1mM phenylmethylsulfonyl fluoride, 20 mM β-glycerophosphate, 1 mM Na_3_VO_4_, and 1 μM okadaic acid). The suspension was incubated for 45 min on ice with occasional mixing, insoluble material was removed by centrifugation (15 min at 12,000 x *g*), and protein concentration in the supernatant was determined using the BioRad protein assay. Equal amounts of extract were subjected to SDS-PAGE and immunoblotting, and band intensity was quantified using an ImageQuant LAS4000 digital imaging system.

### Immunoprecipitation

Cells were lysed in 0.5 mL of lysis buffer (40 mM HEPES, pH 7.4, 120 mM NaCl, 1% Triton X-100, 50 mM NaF, 1 mM EDTA, supplemented with protease and phosphatase inhibitors (EDTA-free complete protease inhibitor tablets, 1% aprotinin, 50 μg/ml leupeptin, 10 μM pepstatin, 1 mM phenylmethylsulfonyl fluoride, 20 mM β-glycerophosphate, 1 mM Na_3_VO_4_, and 1 μM okadaic acid]) by incubating on ice for 45 min and centrifugation at 12,000 x g for 15 min. The extract (1 mg) was precleared with 50 μL agarose beads, according to the manufacturer's directions (Santa Cruz), and to the supernatant was added 5 μg of monoclonal antibody to Mcl-1. After mixing for 3 h at 4°C, the lysates were then incubated with 100 μL of protein A/G PLUS-Agarose beads overnight at 4°C. The beads were pelleted by centrifugation at 1,000 x g for 5 min and washed three times with 0.3 mL of lysis buffer. The beads were resuspended in 100 μL of 2 × SDS loading buffer and incubated for 5 min at 95°C. The immunoprecipitated samples were resolved by 12.5% acrylamide SDS-PAGE gels (Bio-Rad) and analyzed by immunoblotting. Immunoprecipitation of Bcl-xL was performed as described previously [22].

### Preparative immuno-purification of phosphorylated Mcl-1

HeLa cells growing in 14 × 100-mm dishes were synchronized at the G1/S boundary by double thymidine block, treated with 30 nM vinblastine for 15 h, with the final 2 h in the presence of 20 μM MG132, cells were lysed, and 30-35 mg of protein subject to immunoprecipitation with 0.8 mL Mcl-1 antibody and 3 mL of protein A/G PLUS-Agarose beads, as described above. The following steps were carried out essentially as described previously for immunopurification of phosphorylated Bcl-xL [33]. Briefly, washed beads were incubated in 0.5 M NH_4_OH, 0.5 mM EDTA to elute bound proteins, samples were dried in a speed vacuum, dissolved in 80 μL SDS-PAGE sample buffer, resolved by SDS-PAGE using a 15-cm separation gel, and stained with Colloidal Coomassie Blue.

### *In vitro* phosphorylation of Mcl-1

Reactions mixtures of 0.1 mL contained 25 mM Tris-HCl, pH 7.5, 10 mM MgCl_2_, 5 mM DTT, 10 μg His-tagged Mcl-1, 0.1 μg Cdk1/cyclin A2, 1 mM ATP, and 1 μCi [γ-^32^P]ATP. After 2 h at 30°C, reactions were terminated by the addition of EDTA to 20 mM, and subjected to SDS-PAGE. Gels were stained with Colloidal Coomassie Blue, destained, dried and exposed to a phosphoimager screen. For the analysis of Cdk1-catalyzed phosphorylation sites by mass spectrometry, the same reaction was carried out but with the omission of [γ-^32^P]ATP.

### LC-MS/MS methods

Protein gel bands were excised and subjected to in-gel trypsin digestion as follows. Gel slices were destained in 50% methanol, 100 mM NH_4_HCO_3_, followed by reduction in 10 mM Tris[2-carboxyethyl]phosphine and alkylation in 50 mM iodoacetamide. Gel slices were then dehydrated in acetonitrile, followed by addition of 100 ng porcine sequencing grade modified trypsin in 100 mM NH_4_HCO_3_ and incubation at 37°C for 12-16 hours. Peptide products were then acidified in 0.1% formic acid. Tryptic peptides were separated by reverse phase Jupiter Proteo resin (Phenomenex) on a 100 × 0.075 mm column using a nano2D HPLC system (Eksigent) or a nanoAcquity UPLC system (Waters). Peptides were eluted using a 30 min gradient from 97:3 to 35:65 buffer A:B ratio (buffer A = 0.1% formic acid, 0.5% acetonitrile; buffer B = 0.1% formic acid, 75% acetonitrile). Eluted peptides were ionized by electrospray (1.9 kV) followed by MS/MS analysis using collision induced dissociation on an LTQ Orbitrap or LTQ Orbitrap Velos mass spectrometer (Thermo). MS data were acquired using the FTMS analyzer in profile mode at a resolution of 60,000 over a range of 375 to 1500 m/z. MS/MS data were acquired using the ion trap analyzer in centroid mode and normal mass range with a normalized collision energy of 35.0. Proteins and phosphopeptides were identified by database search using Mascot (Matrix Science), with phosphopeptide MS/MS spectra manually verified.

### Site-directed mutagenesis

A plasmid encoding untagged human Mcl-1 in pcDNA3.1 vector served as a template. Serine or threonine to alanine mutants were generated using the QuikChange site-directed mutagenesis kit (Stratagene) according to the manufacturer's instructions. The nucleotide sequences of all cDNA constructs were verified by automated DNA sequencing.

### Two-dimensional polyacrylamide gel electrophoresis

This was carried out essentially as described [23]. Briefly, cells were washed in PBS and resuspended in 7 M urea, 2M thiourea, 4% CHAPS, 2 mM tributylphosphine (TBP), and 1% Omni Cleave^™^ Endonuclease. The suspension was incubated on ice for 30 min, and centrifuged at 4°C at 9,200 x g for 15 min. After determination of the protein concentration, samples were rehydrated with rehydration buffer (7 M urea, 2 M thiourea, 4% CHAPS, 2 mM TBP, 0.2% 5/7 Bio-Lyte Ampholytes, and 0.001% Bromophenol Blue). Isoelectric focusing was carried out in 11-cm pH 5/7 strips for 50,000 Vh. The strips were equilibrated with equilibration buffer (0.375 M Tris-HCl, pH 8.8, 2% SDS, 20% Glycerol, and 5 mM TBP) and SDS-PAGE in the second dimension was performed using 8-16% polyacrylamide gels. After transfer to polyvinylidene difluoride membrane, immunoblotting was performed for Mcl-1.

### Phosphatase treatment

Cell extracts were prepared by lysing cells under nondenaturing conditions in 40 mM HEPES (pH 7.5), 120 mM NaCl, 50 mM NaF, 1% Triton X-100, 1 mM EDTA, 1% aprotinin, 50 μg/mL leupeptin, 1 mM phenylmethylsulfonyl fluoride. Aliquots of 100 μg were incubated with 400 U of lambda protein phosphatase in 25 μL phosphatase buffer (50 mM Tris-HCl, pH 7.5, 0.1 mM EDTA, 5 mM DTT, 0.01% Brij 35, and 2 mM MnCl_2_) at 30°C for 30 min. The samples were precipitated by the addition of trichloroacetic acid to 25% v/v, dissolved in rehydration buffer and analyzed by 2D PAGE/immunoblotting as described above.

### Cell viability assays

Cells (2,000 per well) were seeded in 96-well plates, and vinblastine at half-log increments from 0 to 1 μM was added in a fixed final concentration of 0.1% DMSO. After incubating for 48 h, 3-(4,5-dimethylthiazol-2-yl)-2,5-diphenyltetrazolium bromide (MTT) reagent (50 μg/10 μL/well) was added and incubated overnight at 37°C. The following day, 0.1 mL of 10% SDS in 0.01 M HCl was added, and after overnight incubation, absorbance readings were taken at 540 nm. Each condition was conducted with n = 6, and the data plotted and analyzed using GraphPad Prism software.

### Caspase-3 assay

Caspase-3 activity was determined as described previously [31].

## SUPPLEMENTARY MATERIALS FIGURES



## Abbreviations

MTA: microtubule targeting agent
Cdk: cyclin-dependent kinase
2D-PAGE: two-dimensional polyacrylamide gel electrophoresis
MS: mass spectrometry
