# Supplementary material for: Mitotic arrest-induced phosphorylation of Mcl-1 revisited using two-dimensional gel electrophoresis and phosphoproteomics: nine phosphorylation sites identified
Source: Oncotarget. 2016 Oct 12;7(48):78958–70. doi: 10.18632/oncotarget.12586 (PMC5346690; doi:10.18632/oncotarget.12586)
Supplement: Supplementary file 1 [file oncotarget-07-78958-s001.pdf]

Mitotic arrest-induced phosphorylation of Mcl-1 revisited using two-dimensional gel electrophoresis and phosphoproteomics: nine phosphorylation sites identified

SUPPLEMENTARY FIGURES

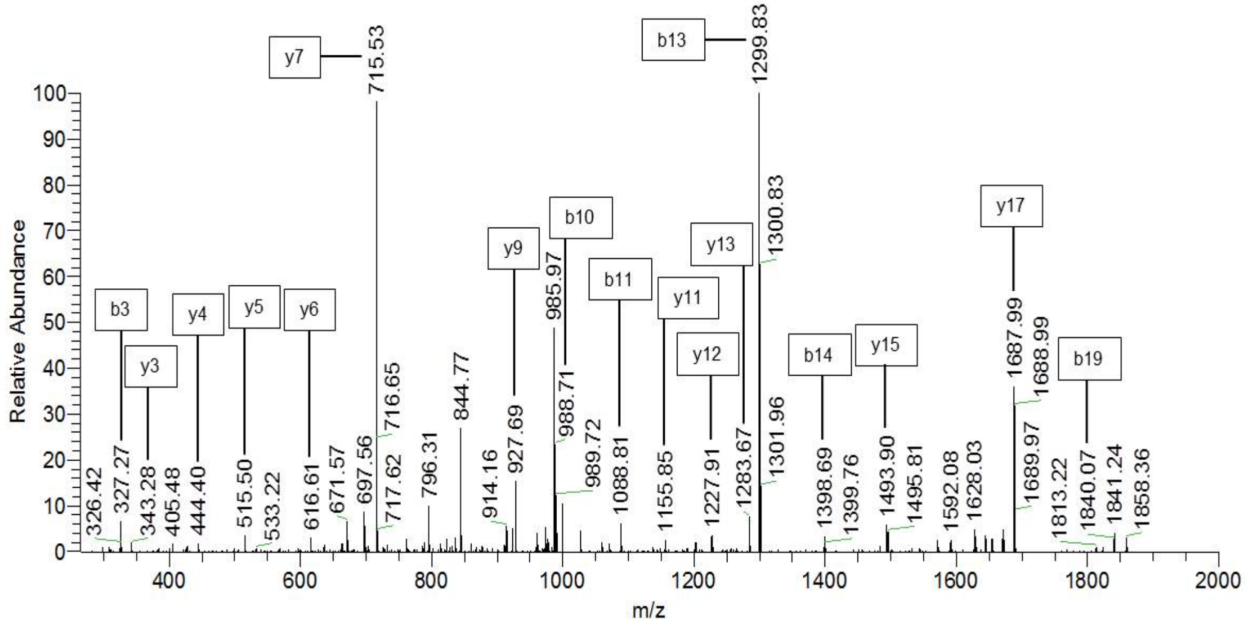

Supplementary Figure S1: MS/MS spectrum of unphosphorylated Mcl-1 peptide VARPPPIGAEVPDVTATPAR (2013.10 Da monoisotopic molecular weight) with prominent b and y ions indicated for comparison with Figure 2B.

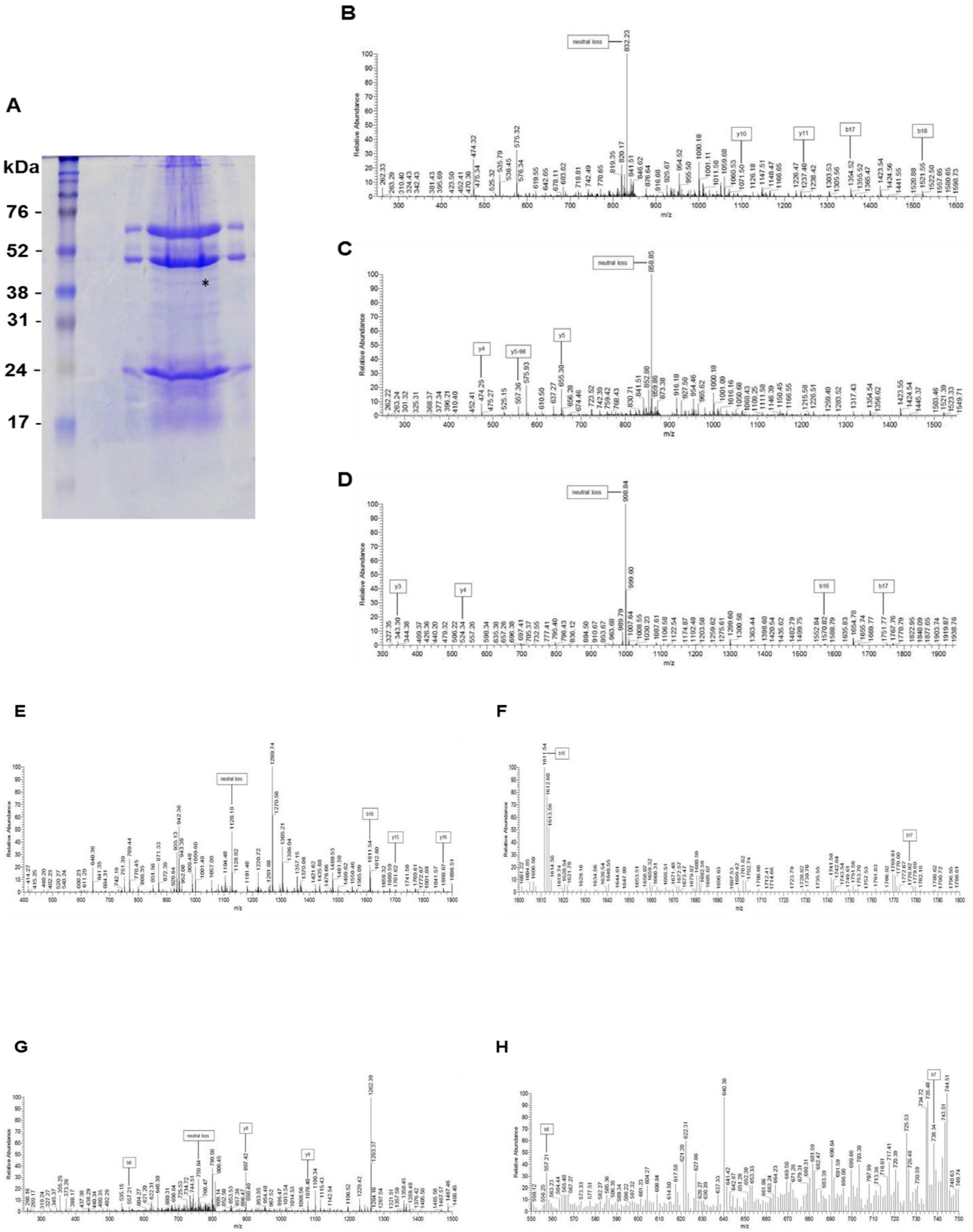

Supplementary Figure S2: Identification of mitotic arrest induced phosphorylation sites in Mcl-1. A. HeLa cells were synchronized at the G1/S boundary, treated with 30 nM vinblastine and 20 µM MG132, and phosphorylated Mcl-1 purified by preparative immunoprecipitation and SDS-PAGE, as described in Materials and Methods. The Coomassie stained band indicated with an asterisk was excised from the gel and subjected to MS/MS analysis. **B.** MS/MS spectrum of Mcl-1 peptide phosphorylated at S64 with neutral loss product and b and y ions defining the phosphorylation site indicated. **C.** MS/MS spectrum of Mcl-1 peptide phosphorylated at T70 with neutral loss product and y ions defining the phosphorylation site indicated. **D.** MS/MS spectrum of Mcl-1 peptide phosphorylated at T92 with neutral loss product and b and y ions defining the phosphorylation site indicated. **E.** MS/MS spectrum of Mcl-1 peptide phosphorylated at S121 with neutral loss product and y ions defining the phosphorylation site indicated. **F.** Detail from E with b ions defining the phosphorylation site indicated. **G.** MS/MS spectrum of Mcl-1 peptide phosphorylated at T163 with neutral loss product and y ions defining the phosphorylation site indicated. **H.** detail from G with b ions defining the phosphorylation site indicated.

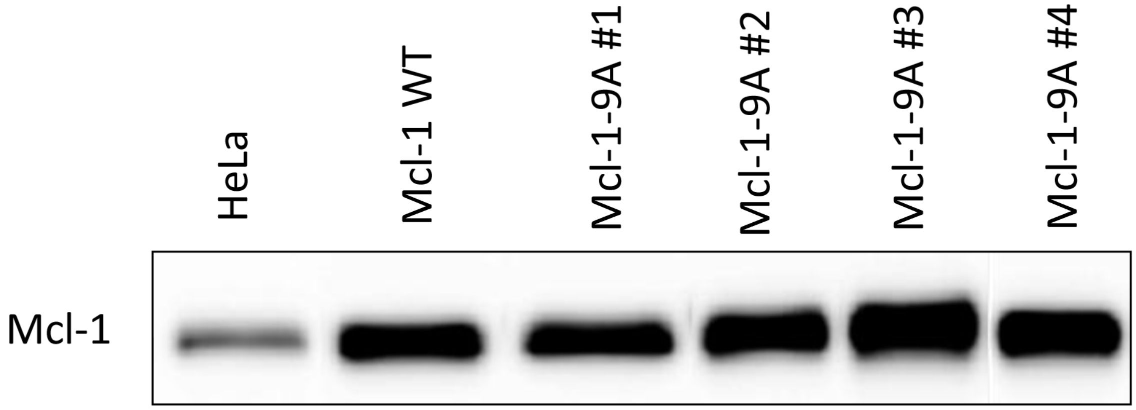

Supplementary Figure S3: Mcl-1 expression in parental HeLa cells or in cells overexpressing wild-type (WT) or 9A mutant Mcl-1. Note that the lanes shown were cropped from a blot that originally contained several other non-relevant lanes.
